# Supplementary material for: Nucleoprotein as a Promising Antigen for Broadly Protective Influenza Vaccines
Source: Vaccines (Basel). 2023 Nov 23;11(12):1747. doi: 10.3390/vaccines11121747 (PMC10747533; doi:10.3390/vaccines11121747)
Supplement: Supplementary file 1 [file vaccines-11-01747-s001.zip › vaccines-2679436-supplementary.pdf]

vaccines

Supplementary Materials

```
           10         20         30         40         50         60         70         80         90        100        110
1 :MASQGTKRSY EQMETDGERQ NATEIRASVG KMIGGIGRFY IQMCTELKLS DYEGRLIQNS LTIERMVLSA FDERRNKYLE EHPSAGKDPK KTGGPIYRRV NGKWMRELIL
2 :.......... .......... .......... ...D...... .......... .......... .......... .......... .......... .......K.. D.......V.
3 :.......... .......D.. .......... ...D...... .......... .H........ ....K..... .......... .......... .......... D.......V.
4 :.......... .....G.... D......... R......... .......... ..D....... I......... .......... .......... .......... G.........
5 :.......... .....G.... DT........ R......... .......... ..D....... I......... .......... .......... .........I D...T.....

          120        130        140        150        160        170        180        190        200        210        220
   YDKEEIRRIW RQANNGDDAT AGLTHMMIWH SNLNDATYQR TRALVRTGMD PRMCSLMQGS TLPRRSGAAG AAVKGVGTMV MELVRMIKRG INDRNFWRGE NGRKTRIAYE
   .......... .......... .......... .....T.... .......... .......... .......... .......... ...I...... .......... ......S...
   .......... ......E... S......... .......... .......... .......... .......... .....I.... ...I...... .......... ......S..D
   ........V. ......E... .....I.... .......... .......... .......... .......... ........IA ...I...... .......... ...R..V...
   ........V. ......E... .....I.... .......... .......... .......... .......... ........IA ...I...... .......... ...R..V...

          230        240        250        260        270        280        290        300        310        320        330
   RMCNILKGKF QTAAQKAMMD QVRESRNPGN AEFEDLTFLA RSALILRGSV AHKSCLPACV YGPAVASGYD FEREGYSLVG IDPFRLLQNS QVYSLIRPNE NPAHKSQLVW
   .......... .....R.... .......... ..I...I... .......... .......... .......... ..K....... ....K..... .......... ..........
   .......... .....R..V. .......... ..I...I... .......... .........A .....S.... ..K....... ....K..... .I........ ..........
   .......... .....R.... .......... ..I...I... .......... .......... ..L.....H. .......... ....K..... ..V..M.... ..........
   .......... .....R.... .......... ..I...I... .......... .......... ..L.....H. .......... ....K..... ..V..M.... ..........

          340        350        360        370        380        390        400        410        420        430        440
   MACHSAAFED LRVLSFIKGT KVLPRGKLST RGVQIASNEN METMESSTLE LRSRYWAIRT RSGGNTNQQR ASAGQISIQP TFSVQRNLPF DRTTIMAAFN GNTEGRTSDM
   ...N...... .......R.. ..S....... .......... .DA....... .......... .......... .......V.. A......... .KP......T ..........
   .......... ..L....R.. ..S....... .......... .DN.G..... ...G...... .......... .....T.V.. .......I.. EKS......T ..........
   .......... ...S...R.K ..I....... .......... V...D.N... .......... .........K .......V.. .......... E.A.V....S ..N.......
   .......... ...S...R.K ..I....... .......... V...D.N... .......... .........K .......V.. .......... E.A......S ..N.......

          450        460        470        480        490        500
   RTEIIRMMES ARPEDVSFQG RGVFELSDEK AASPIVPSFD MSNEGSYFFG DNAEEYDN
   .A.......G .K..EM.... .......... ..N....... .......... ........
   .A.......G .K..E...R. .......... ..N....... .......... ........
   ...V...... .K...L.... .......... .TN....... .......... .......S
   ...V...... .KQ..L.... .......... .TN....... .......... ........
```

1 – A/PR8/8/34 (H1N1)
2 – A/Hong Kong/01/1968 (H3N2)
3 – A/Darwin/9/2021 (H3N2)
4 – A/California/7/2009 (H1N1)
5 – A/Victoria/4897/2022 (H1N1)

Figure S1. Alignment of NP sequences of A/PR/8/34 (H1N1) influenza virus and recent H1N1 and H3N2 seasonal influenza strains. The figure was generated using GISAID data and UGene software.

```
           10         20         30         40         50         60         70         80         90        100        110
1 :MASQGTKRSY EQMETDGERQ NATEIRASVG KMIDGIGRFY IQMCTELKLS DYEGRLIQNS LTIERMVLSA FDERRNKYLE EHPSAGKDPK KTGGPIYKRV DGKWMRELVL
2 :.......... .......... .......... .......... .......... .......... .......... .......... .......... .......... ..........
3 :.......... .......D.. .......... .......... .......... .H........ ....K..... .......... .......... .......R.. ..........
4 :.......... .....G.... D......... R..G...... .......... ..D....... I......... .......... .......... .......R.. G.......I.
5 :.......... .....G.... DT........ R..G...... .......... ..D....... I......... .......... .......... .......R.I ....T...I.

          120        130        140        150        160        170        180        190        200        210        220
   YDKEEIRRIW RQANNGDDAT AGLTHMMIWH SNLNDTTYQR TRALVRTGMD PRMCSLMQGS TLPRRSGAAG AAVKGVGTMV MELIRMIKRG INDRNFWRGE NGRKTRIAYE
   .......... .......... .......... .......... .......... .......... .......... .......... .......... .......... ......S...
   .......... ......E... S......... .....A.... .......... .......... .......... .....I.... .......... .......... ......S..D
   ........V. ......E... .....I.... .....A.... .......... .......... .......... ........IA .......... .......... ...R..V...
   ........V. ......E... .....I.... .....A.... .......... .......... .......... ........IA .......... .......... ...R..V...

          230        240        250        260        270        280        290        300        310        320        330
   RMCNILKGKF QTAAQRAMMD QVRESRNPGN AEIEDLIFLA RSALILRGSV AHKSCLPACV YGPAVASGYE FEKEGYSLVG IDPFKLLQNS QVYSLIRPNE NPAHKSQLVW
   .......... .......... .......... .......... .......... .......... .........D .......... .......... .......... ..........
   .......... .......V.. .......... .......... .......... .........A .....S...D .......... .......... .I........ ..........
   .......... .......... .......... .......... .......... .......... ..L.....HD ..R....... .......... ..V..M.... ..........
   .......... .......... .......... .......... .......... .......... ..L.....HD ..R....... .......... ..V..M.... ..........

          340        350        360        370        380        390        400        410        420        430        440
   MACNSAAFED LRVSSFIRGT KVIPRGKLST RGVQIASNEN MDTMESSTLE LRSRYWAIRT RSGGNTNQQR ASAGQISVQP TFSVQRNLPF DKPTIMAAFT GNAEGRTSDM
   .......... ...L...... ..S....... .......... ..A....... .......... .......... .......... A......... .......... ..T.......
   ...H...... ..LL...... ..S....... .......... ..N.G..... ...G...... .......... .....T.... .......I.. E.S....... ..T.......
   ...H...... .........K .......... .......... VE..D.N... .......... .........K .......... .......... ERA.V....S ..N.......
   ...H...... .........K .......... .......... VE..D.N... .......... .........K .......... .......... ERA......S ..N.......

          450        460        470        480        490        500
   RAEIIRMMEG AKPEEVSFQG RGVFELSDEK ATNPIVPSFD MSNEGSYFFG DNAEEYDN
   .......... .....M.... .......... .A........ .......... ........
   .......... ........R. .......... .A........ .......... ........
   .T.V.....S ....DL.... .......... .......... .......... .......S
   .T.V.....S ..Q.DL.... .......... .......... .......... ........
```

1 – A/Leningrad/134/17/57 (H2N2)
2 – A/Hong Kong/01/1968 (H3N2)
3 – A/Darwin/9/2021 (H3N2)
4 – A/California/7/2009 (H1N1)
5 – A/Victoria/4897/2022 (H1N1)

Figure S2. Alignment of NP sequences of A/Leningrad/134/17/57 (H2N2) influenza virus and recent H1N1 and H3N2 seasonal influenza strains. The figure was generated using GISAID data and UGene software.
